# Editorial: Endothelial activation and microcirculatory disorders in sepsis and critical illness

**DOI:** 10.3389/fmed.2022.1133408

**Published:** 2023-01-11

**Authors:** Jérémie Joffre, W. Conrad Liles

**Affiliations:** ^1^Medical Intensive Care Unit, Hôpital Saint Antoine, Sorbonne University, Assistance Publique-Hôpitaux de Paris, Paris, France; ^2^Centre de Recherche Saint-Antoine (CRSA), INSERM UMR_S938, Paris, France; ^3^Department of Medicine (Primary), Laboratory Medicine, Pharmacology, and Global Health, University of Washington, Seattle, WA, United States; ^4^Department of Pathology, Laboratory Medicine, Pharmacology, and Global Health, University of Washington, Seattle, WA, United States; ^5^Sepsis Center of Research Excellence-UW (SCORE-UW), University of Washington, Seattle, WA, United States; ^6^Center for Lung Biology, University of Washington, Seattle, WA, United States

**Keywords:** endothelium, microcirculation, critical illness, sepsis, glycocalyx (glycocalix)

Over the past two decades, clinical and experimental studies have shown that endothelial and microvascular dysfunction are key drivers of organ failure in critical illnesses, such as sepsis, cardiogenic shock, and trauma. From the basic mechanisms of endothelial activation and signaling under inflammatory conditions to clinical trials targeting microcirculation, this field of research is one of the most dynamic and promising in the critical care community.

Therefore, we, as co-guest editors, are pleased to present the first volume of the topic collection “*Endothelium and Microvascular Dysfunction in Critical Illnes*s” in *Frontiers in medicine*. This first volume, broad in scope, aimed to cover the multiple facets of endothelial biology and its translational opportunities. It starts with a general review by Raia and Zafrani. and a narrative review specifically focused on glycocalyx (GCX) biology (Patterson et al.) and the potential pharmacological strategies to protect or restore it during critical illness and injury. Nevertheless, assessing GCX integrity is challenging in daily practice. To do so, Bol et al. reported a remarkable study about multimodal assessment of GCX degradation during CABG combining SDF GCX thickness assessment by sublingual capillaroscopy and biomarkers of GCX degradation and EC activation. They highlighted a significant correlation between GCX thinning during CABG and elevation of GCX shedding biomarkers, but not associated with biomarkers of permeability such as Tie-2 and Ang-2 (Bol et al.).

Several original research articles accepted in the collection are remarkable and complementary: the study by Amunugama et al. provides new insight into the microvascular biology during sepsis by showing that endothelial cell lipidomics is differentially altered depending on *Escherichia coli* strain infection and interactions with neutrophils, suggesting that despite a pleiotropic and redundant system, the endothelial response to bacterial pathogens varies differentially in terms of the pro-adhesive and/or pro-coagulant phenotype induced by a specific pathogen. This result is supported by the study of Dechamps et al. that performed a differential analysis of the coagulopathy present in patients with sepsis vs. severe COVID-19, suggesting that inflammation-induced endothelial activation and coagulopathy differs substantially from sepsis-induced coagulopathy.

Among the articles with a primary focus on COVID-19, Gao et al. reported that COVID-19 patients may experience long-lasting endothelial dysfunction up to 10 months after recovery, characterized by an altered flow-mediated vasodilation response and associated with low-grade persisting inflammation. Such findings are significant regarding the potentially delayed cardiovascular adverse events experienced by ICU survivors as part of the post-ICU syndrome (1, 2).

Despite accumulated evidence that endothelial dysfunction and microcirculatory failure are key factors in determining clinical outcome and recent advances in microcirculation monitoring, integrating the microcirculation or the endothelium into resuscitation strategies or adjuvant therapy algorithms remains highly challenging. In this Research Topic, van Lier et al. performed a *post-hoc* analysis of the AdrenOSS-2 study (3). The AdrenOSS-2 study was a phase 2a, randomized placebo-controlled clinical trial using a non-neutralizing anti-adrenomedullin antibody in early septic shock patients with serum adrenomedullin at admission > 70 pg/ml. This work was a proof of concept and dose-finding study, documenting feasibility of biomarker-guided therapy and the safety of adrecizumab. The authors reported that a cDPP3-based enrichment strategy may help to identify a sub-group of patients with sepsis in which the effect of adrecizumab is more pronounced. Specifically, in the sub-group of patients with admission cDPP3 < 50 ng/ml that received early treatment, the impact of adrecizumab on decreasing organ failure at 24 h (delta SOFA) was significant (*p* = 0.045), and the increase in survival was close to significant (HR:0.49 [0.2–1.18]; *p* = 0.094) (van Lier et al.). The study illustrates that innovative precision medicine strategies based on endothelial-related biomarkers may improve clinical outcomes in septic shock.

Building on the success of *Endothelial Activation and Microcirculatory Disorders in Sepsis and Critical Illness*, volume I collection, we are pleased to launch the volume II of this Research Topic. Therefore, we welcome submissions of Original Research, Review, and Correspondence focusing on the following areas of research:

- Basic endothelial biology focusing on the immune role of the endothelium, coagulation/fibrinolysis, oxidative stress, leukocytes/ platelets adhesion, rheology, vasoreactivity or endothelial heterogeneity;
- Biomarker and translational studies;
- Assessment and monitoring of microcirculatory disorders in critical illness or major surgery;
- Bioinformatic and digital solutions for real-time multiparametric hemodynamic assessment and/or outcome prediction.

## Author contributions

All authors listed have made a substantial, direct, and intellectual contribution to the work and approved it for publication.
